# On the Urinary Stones and Gravel in Men

**Published:** 1801-12

**Authors:** 


### *On the Urinary Stones and Gravel in Men.*
### *By* Cit. Fourcroy.

IT is only fince the time of *Scheele* that we have become acquainted with the nature of urinary calculi, this fubject having been quite in the dark before that great chemift difcovered, in the year 1776, a peculiar acid (the lithic acid) in them, and at the fame time found them to contain no lime, a circumftance which was foon after confirmed by the experiments of *Bergmann.* From this period, the chemifts beftowed a particular attention upon the examination of urinary concrements, as appears from the writings of *Dobson*, *Percival*, *Falconer*, *Achard*, *Hartenkeit*, *Tychsen*, *Link*, *Titius*, *Walther*, *Gartner*, *Brugnatelli*, *Pearson*, and feveral others, fome of whom confirmed the difcovery of *Scheele*, while others contradicted, and others enlarged it.

But we are particularly indebted to *Cit. Fourcroy* and *Vauquelin*, who fince 1786 had turned their attention on this fubject, for having made many experiments, by which great light is thrown on the nature of urinary concrements. We have thought it proper to communicate to our readers in an extract, the interefting refults of their chemical inquiries, principally on account of the influence they have on the treatment of urinary calculi.

Section I. *On the seat and physical properties of Urinary Calculi.* — Calculi are found in different parts of the urinary ſyſtem, in the pelvis renalis, in the ureters, in the bladder and urethra; but as they, for the moſt part, originate in the pelvis renalis, the calculi renales make the nucleus of the greateſt number of urinary ſtones. The *calculi renales* differ greatly with reſpect to their external qualities; for the moſt part, however, they conſiſt of ſmall, concrete, roundiſh, ſmooth, gloſſy, and cryſtalline bodies, of a red-yellow colour, like that of wood, and ſo hard as to admit poliſhing. On account of their minuteneſs, they eaſily paſs through the urinary paſſages in form of gravel, which being ſometimes of a rough ſurface, cauſe ſeveral complaints on their paſſage. But in ſome inſtances they are of too great a ſize to be able to penetrate through the ureters; in which caſe they increaſe in the kidneys, to the manifeſt injury of them. Calculi renales of this kind are generally of a brown, dark red, or black colour, and ſurrounded with ſeveral ſtrata of coagulated blood and pus; they have alſo been obſerved of a yellow, reddiſh, and lighter colour; and ſome conſiſting of an homogenous ſtony maſs: but white or grey calculi renales are very rarely to be met with. Amongſt the great number that were examined by me, I have only found one or two of a grey or blackiſh colour, and a compoſition ſimilar to thoſe which generally bear the name of mulberry-like ſtones.

*The stones in the ureters* are calculi renales, which on paſſing into the ureters are prevented by their ſize from deſcending into the bladder, and frequently increaſe very much; they, however, rarely occur, but ſtill rarer are the internal ſtony coverings of the ureters, which entirely obſtruct the paſſage of urine; their colour is white, and they conſiſt of phoſphat of lime.

*The stones in the bladder* are the moſt frequent urinary concrements that have been principally examined; they draw their firſt origin from the kidneys, whence they deſcend into the bladder, where they increaſe, or they immediately originate and increaſe in the bladder; or they ariſe from a foreign body that by chance has got into the bladder, which not unfrequently happens, particularly in the female ſex. Concrements of this kind differ greatly in their reſpective phyſical qualities and external form, which, however, is generally ſpherical, oval, or compreſſed on both ſides; and ſometimes, when there are ſeveral ſtones in the bladder, they have a polyedrous or cubical form; their extremities are frequently pointed or roundiſh, but they are very ſeldom found cylindrical, and more rarely with cylinders at their ends.

There is a great variety in the ſize of the calculi, and likewiſe wife in their colour, which is materially different according to their refpective nature and compofition. They occur, 1. of a yellowifh colour, like wood, approaching nearly to red or brown; fuch ftones confift of lithic acid. 2. Grey, or more or lefs white; thefe ftones always contain phofphats of earths. 3. Dark grey or blackifh; ftones of this colour have oxalats of earths. Many ftones fhow brown or grey fpots on a yellow or white ground, generally raifed on the furface, and confifting of oxalat of lime, which is enclofed in lithic acid when the ground colour of the ftone is of a wood colour, or in phofphat of lime when it is white. Thefe fpots are, in general, only to be obferved in the middle of the ftone, or at one of its extremities. All that I have here ftated is the refult of obfervations on more than 600 calculi; and different other colours that are faid to have been obferved, either arife from heterogeneous fubftances, or are merely variations of the above colours. Their furface is fmooth and polifhed in fome, in others only fmooth, and in others uneven, and covered with rough or fmooth corpufcles, which are always of a yellow colour; in fome the furface is partly fmooth, and partly rough. The white ones are frequently even and fmooth, half tranfparent, and covered with fhining cryftals, that generally indicate phofphat of ammonia with talc; or they are faint, and confift of minute grains; or rough, in which cafe they confift of phofphat of lime. The brown and dark grey ftones are, from their fimilarity to mulberries, called mulberry-ftones, and being frequently very rugged, they caufe the moft pain of all.

On examining the fpecific weight of urinary calculi in more than 500 fpecimens, I found it to be in the lighteft 1213: 1000, in the heavieft as 1976:1000. Their fmell is partly ftrong, like urine or ammonia, partly infipid, and terreous; for inftance, the white ones, which are like fawed ivory or rafped bone.

The internal texture of calculi is but feldom gueffed from their external appearance, particularly when they exceed the fize of a pigeon's egg. On breaking them they generally feparate into two or three ftrata, more or lefs thick and even, which prove that they are formed by different precipitations at different times. In the middle a kernel is generally feen, of the fame mafs as the reft. When the place they are broken at is finely ftreaked, and of a yellow or reddifh colour, the lithic acid predominates; but when they are half tranfparent, luminous like fpar, they have ammoniacal magnefia in them, and phofphat of lime when they are brittle and friable; but when they are fo hard as to refift the inftrument, of a fmooth furface, and a fmell like ivory, they contain faccharic lime. It frequently happens, that the exterior ftratum confifts of white phofphat phoſphat of earth, while the kernel is yellow lithic acid or oxalat of lime, covered ſometimes with a yellow ſtratum of lithic acid, in which caſe the kernel appears radiant; but when it conſiſts of lithic acid, and is covered with white phoſphate of earth, it is roundiſh, oval, and ſomewhat crooked. Theſe concrements have very ſeldom three ſtrata; namely, on the outſide a phoſphat of ſalt, towards the inſide lithic acid, and quite within ſide an oxalat of lime; but ſtill rarer theſe ſubſtances occur in more ſtrata or in another order, as before mentioned. Stones of the urethra are ſeldom generated in the urethra itſelf; however, there are inſtances of their having been formed in the foſſa navicularis by means of foreign bodies that have got into the urethra. We alſo very frequently obſerve ſtony concrements depoſited between the glans and prepuce. All the concrements produced within ſide and outſide of the urethra conſiſt of phoſphats of earths, which are eaſily precipitated from the urine. There are likewiſe ſtones in the urethra, which have come out of the bladder, having been produced there, or in the kidneys, and they generally poſſeſs the properties of ſtones of the kidneys.

Sect. II. *On the different constituent particles of urinary stones.* It has been mentioned above that Scheele found a peculiar acid in the urinary concrements, and likewiſe that phoſphat of lime was diſcovered in them. The identity of the lithic acid, however, was much doubted by modern chemiſts, particularly by *Pearson*, who aſſerted that it was merely an oxyd, whereby he gave riſe to the diſcoveries Mr. *Vauquelin* and myſelf have ſince made on this ſubject, becauſe we were induced to repeat the experiments which we had propoſed to the public, in order to examine whether the lithic acid be really an acid, or to confirm our former opinion of it. Our endeavours were fully rewarded, as we not only found the lithic acid and phoſphat of lime in the different calculi, but alſo five other ſubſtances, viz. the lithat of ammonia, oxalat of lime, ſiliceous earth, phoſphat of ammoniacal magneſia, and an animal matter.

1. *Of the lithic or uric acid.* The acid diſcovered by Mr. Scheele in the urinary concrements was ſtiled lithic acid; or, according to Dr. Pearſon's reſearches, uric acid, which, after Scheele, has the following properties. It is inſipid, without ſmell, hard, cryſtallizible, not ſoluble in cold water, and in boiling water only in ſeveral thouſand times greater quantity. This ſolution, after having become cool, depoſits the acid in form of minute yellow needles, eaſily ſoluble in the lye of fixed alkalis, out of which, however, it is precipitated by all acids, even the carbonic acid, except the ſulphuric and muriatic acid, which have no effect on it. Concentrated nitric acid, on diſ-

folving it, obtains a red colour. On diftilling the lithic acid it yields a fmall quantity of fublimed, undecompofed uric acid, very little oil and water, cryftallized carbonat of ammonia, carbonic acid, and a very black coal, which, however, contains neither alkali nor lime. Befides thefe properties, it poffeffes ftill others, according to our refearches. On rubbing it with concentrated lye of kali or natron, it immediately forms a faponaceous, thick, and pulpy mafs, which is very foluble in water when fuperfaturated with alkali, but little foluble when only faturated with it. The faturated combinations have little tafte, are not cryftallizable, and when diluted with water, the muriatic acid precipitates the uric acid in form of fmall, needle-like, fhining, fomewhat yellowifh cryftals. Ammonia receives very little of it, which combination is almoft quite indiffoluble. Lime water has likewife very little effect on it, and the carbonats of alkalis none at all. On being diffolved in nitric acid, a part of the lithic acid is changed into oxalic acid. The red colour which appears after this combination is faid to prove, according to Pearfon, that fubftance to be merely an oxyd, but it arifes from a peculiar animal matter. When oxygenated muriatic acid is brought in contact with lithic acid, the colour of it grows pale, it puffs up, becomes foft and gelatinous, and at laft obtains the confiftency of a milky liquor; from which procefs only $\frac{1}{60}$ of a white, light, animal fubftance remains, and a quantity of carbonic acid evolves itfelf under continual flow effervefcence. The liquor yields muriat of ammonia, oxalat of ammonia, both in cryftals, free muriatic and malic acid; confequently the oxygenated muriatic acid feparates the uric acid into ammonia, carbonic acid, oxalic acid, and malic acid, whereby we obferve that the oxygenated muriatic acid changes the uric acid, firft into ammonia and malic acid, but on the addition of more acid, into oxalic acid; and when ftill more acid is added, into water and carbonic acid. The remaining white fubftance is the fame, from which the red colour originates that appears on the combination of the uric acid with nitric acid, and which imparts the cubical form to the muriat of ammonia, obtained by the evaporation of the liquor. It remains now to be ftated what is obferved in the diftillation of that acid, at which it yields not only carbonat of ammonia, but alfo carbonic gas, very little oil, Pruffic acid, partly in form of gas, partly in fluid form, a confiderable quantity of coal that contains no falt, and a little water. The productions thus obtained have the fmell of bitter almonds. The refults of thefe inquiries manifeftly fhow, that the lithic acid is really an acid of its own, confifting of azote, carbon, hydrogen, and oxygen. This peculiar acid is an excrementitious fubftance, which which is carried off by the urine, and at the forming of calculi combines itself with a coloured animal matter, from which alfo it probably originates by a procefs ftill unknown.

2. *Of the lithat of ammonia.* This fubftance feems to have been unknown before, or at leaft not properly difcerned from the uric acid, and though Scheele has obferved it, he was ignorant of its particular nature. It is eafily to be diftinguifhed by the fmall even ftrata in which it is formed, by its colour, that looks like milk coloured with coffee, and by its forming but fmall calculi. It diffolves in the lees of kali and natron like the lithic acid, but with the characteriftic difference that it difcharges ammonia, a phenomenon already obferved by Scheele. It is more foluble in cold as well as warm water, than the lithic acid. It is in the fame way affected by acids, except that a greater quantity is required for changing it. It is generally mixed with phofphat of ammoniacal magnefia, becaufe it feems only to take place after a fufficient quantity of ammoniacal magnefia has been formed, to faturate the phofphat of kali and the free uric acid.

3. *Of the phosphat of lime.* The exiftence of this fubftance had hitherto been but inaccurately determined, every fubftance which was not lithic acid being formerly comprifed by the name of phofphat of lime. It occurs in fmall friable ftrata, which break in fcales or fplints of a grey white colour, and are faint, opaque, without any fmell or tafte, and cryftallized in a luminous or fpar-like form; inftead of ftrata it is frequently compofed of friable grains, that flightly cohere, and has many holes and pores like a fpongy texture. It never forms a calculus by itfelf, being in a calculus always united with an animal gelatinous matter; on account of which circumftance it becomes black by expofing it to a ftrong heat, and burns to coal, exhaling the odour of burned bones; and yields water, oil, carbonat of ammonia, and a carbonaceous refiduum. Being calcined white, it only leaves lime, and phofphat of lime, without any water of cryftallization. It is not foluble in cold water, but in boiling water a part of its gelatine diffolves, fpreading an animal odour. All acids, except the boracic and carbonic acid, diffolve it, leaving on the bottom of the veffels tranfparent fpots of animal matter. Thefe folutions are all precipitated by alkalis, but without any decompofition, the precipitation remaining phofphat of lime. On treating the phofphat of lime with concentrated nitric acid, a thick pulpy mafs of acid fulphat and phofphat of lime will be obtained, on which pure alkalis, as well as carbonat of alkalis, have no effect. We never could find acid phofphat of lime, as Brugnatelli pretends to have obferved.

4. Of

4. *Of the phosphat of ammoniacal magnesia.* It consists of scaly, half-transparent, hard and coherent strata; can be sawed without crumbling, and reduced to a fine soft and white powder. It is of a sweetish insipid taste, somewhat soluble, and crystallized in rhomboids, or thick laminas, dispersed in the cavities of other calculous substances; and it is frequently found on the surface of other concrements. It contains betwixt its strata a gelatinous substance, but less than the phosphat of lime, on which account it also blackens by being heated. Though it be but little soluble in water, yet it dissolves in such a quantity as to be capable of crystallizing by slow evaporation. Acids dissolve it more quickly than they do the phosphat of lime. Weak sulphuric acid entirely dissolves it, forming sulphat of ammoniacal magnesia. In diluted muriatic or nitric acid, it disappears more quickly than phosphat of lime. Ammonia, by which that salt is made turbid, only precipitates small particles of magnesia. The lees of fixed alkalis disengage from it ammonia, without forming with it a solution; and depriving it of the phosphoric acid, leave the magnesia behind.

5. *Of the oxalat of lime.* It is, according to our observations, only found in the mulberry-like calculi, in combination with a coloured animal matter, and consist of strata covered with pointed, roundish, rough or smooth protuberances; outside it appears of a dark or brown colour, but internally it is grey, frequently with white streaks, of a solid texture, and may be polished like ivory; it breaks in scales, or in the shape of shells; and, on being pounded or sawed, it exhales an animal odour like semen. It is the heaviest of all calculous substances, and the only one which yields one-third of lime by calcination. It dissolves with difficulty in acids, and is precipitated unaltered by alkalis from nitric acid. The fixed alkalis decompose it when they are impregnated with carbonic acid, and when it is pulverized, and the solution heated, whereby carbonat of lime and oxalat of alkalis are obtained.

The great quantity of animal matter which constantly adheres to this oxalat of lime is very characteristical, which imparts the brown, reddish, blackish colour to the above kind of stones, and likewise the fine and solid texture. This substance may be obtained by putting small pieces of these stones into diluted nitric acid, whereby it appears of the same colour, and becomes soft and spongy. The great hardness of this kind of calculi, most probably arises from the intimate connexion of its particles, produced by the combination of the oxalat of lime with that animal matter, in the same way as lime obtains a great degree of solidity by its combination with albuminous matter, of which, and of a peculiar matter of urine, that animal ſubſtance ſeems to conſiſt.

6. *Of the siliceous earth.* Amongſt 600 calculi which we examined, there were only two that contained this earth; both had the texture of mulberry-like ſtones, though of a lighter colour, and by being calcined loſt one-third of their weight, without giving free lime; heated with acids they loſt nothing, but when melted with four times as much of alkali, they yielded ſiliceous earth by being treated with muriatic acid. They contained phoſphat of lime, and an animal matter ſimilar to that which is united with the oxalat of lime. They were hard, difficult to be ſawed and pulveriſed, and the powder made ſcratches in metal. On being burnt they emit an animal odour; they imparted nothing to the boiling water, and to the acids a little phoſphat of lime, which difficultly ſeparates from the ſiliceous earth. Alkalis, either pure, or combined with carbonic acid, did not affect them, merely depriving them of a part of their animal matter. Their eſſential character conſiſts in their being fuſible and vitrifiable with fixed alkalis.

7. *Of the animal matter.* All the ſix ſubſtances juſt examined, which conſtitute the urinary ſtones of the human ſpecies, are always combined with an animal matter, as appears from its being burnt to coal, from the productions it yields by diſtillation, from its ſtench on being burnt, and from the cellulous membranous floccula which remain when pieces of calculi are diſſolved in diluted acids. This animal matter has been frequently, and with good reaſon, conſidered as the baſis of all urinary concrements, like as in bones the gelatinous matter, the firſt baſis of the bones, forms an organic texture, in the interſtices of which the phoſphat of lime is depoſited. It is very remarkable, that the different conſtituent particles of urinary ſtones are combined with a diſſimilar animal matter, which is ſometimes albuminous, ſometimes gelatinous, ſometimes compoſed of both, and frequently united with the matter of urine. Thus the lithic acid, or the lithat of ammonia, contains a third of albuminous matter, combined with the matter of urine, the phoſphats of earths, albuminous matter, gelatina in form of membranes, and laminas, or tela celluloſa; the oxalat of lime, a ſpongy, yet more ſolid texture of the colour of albumen, and the ſiliceous earth, a ſimilar ſubſtance. On the whole, the animal matter ſeems to unite and join together all the acid and ſaline particles of urinary concrements.

